# A soluble activator that favors the ex vivo expansion of CD8^+^CD27^+^ T cells

**DOI:** 10.1172/jci.insight.141293

**Published:** 2020-11-19

**Authors:** Esther I. Matus, Amanda Sparkes, Jean Gariépy

**Affiliations:** 1 Sunnybrook Research Institute,; 2 Department of Medical Biophysics, and; 3 Department of Pharmaceutical Sciences, University of Toronto, Toronto, Canada, USA.

**Keywords:** Therapeutics, Cancer immunotherapy, T cells

## Abstract

Adoptive cell therapy involves the infusion of tumor-reactive T cells into patients with cancer to provide antitumor immunity. The ex vivo expansion and differentiation of such T cells are key parameters that affect their therapeutic potential. Human T cells are presently expanded in culture through the use of anti-CD3 and anti-CD28 mAbs immobilized on beads, expressed on cells, or assembled in the context of soluble antibody complexes. Here we report the design of a small, bispecific single-chain variable fragment construct agonizing both CD3 and CD28 pathways. This soluble T cell expansion protein, termed T-CEP, activates, expands, and differentiates human T cells ex vivo at concentrations in the femtomolar range. Importantly, T-CEP promotes the preferential growth of human CD8^+^ T cells over the course of 12 days in comparison with methods involving immobilized anti-CD3 mAb/soluble anti-CD28 mAb or soluble anti-CD3/CD28 mAb complexes. The differentiation profile of the resulting human T cell population is also singularly affected by T-CEP, favoring the expansion of a preferred CD8^+^CD27^+^ T cell phenotype. The activity profile of T-CEP on human T cells ex vivo suggests its use in generating human T cell populations that are more suited for adoptive cell therapy.

## Abstract 2

A bispecific protein construct is described that rapidly expands primary human T cell populations ex vivo at femtomolar concentrations with desirable phenotypic properties for adoptive cell therapy.

## Introduction

Adoptive cell therapy (ACT) regroups cell-based clinical approaches typically exemplified by lymphocytes with antitumor activity being transferred into patients to eliminate specific cancer cells (1). Such approaches have been successful in treating hematological malignancies, metastatic melanoma, and metastatic breast cancer (2–4). Depending on the type of malignancy, treatment may be carried out using a patient’s own tumor-infiltrating lymphocytes (TILs), by designing chimeric antigen receptor (CAR) T cells, or by employing genetically modified T cells. These lymphocytes, and in particular highly functional human T cells, must all be expanded ex vivo before infusion into a patient (5–7), a key step that requires overcoming challenges associated with expanding such cells to generate clinically effective doses (7). Important steps for the ex vivo activation, expansion, and survival of human T cells include a TCR-mediated activation signal, a costimulatory signal, and signaling through cytokines (8). In vitro polyclonal T cell activation is generally accomplished through the use of agonistic antibodies targeting CD3 and CD28 providing both TCR activation and costimulatory signals, respectively, with appropriate cytokines being supplemented (5).

There are several clinically important criteria linked to the success of using human T cells for ACT. First, CD8^+^ T cells are favored in ACT due to their cytolytic activity (9), although transferred CD4^+^ T cells also display the potential to enhance CD8^+^ T cell–mediated tumor rejection (10). A second key parameter is the differentiation state of the resulting T cell product expanded ex vivo. Specifically, T cell differentiation events take place during their expansion, which can ultimately lead to a loss of crucial ACT characteristics required for their optimal persistence and function in vivo (11, 12). Less differentiated T cells are considered more desirable for ACT, which can be distinguished based on the surface expression of known markers for naive T cells (Tn), stem cell memory T cells (Tscm), and central memory T cells (Tcm) as opposed to more differentiated effector memory T cells (Tem) and terminally differentiated T cells (Temra) (13, 14). T cell activation, proliferation, and differentiation through agonizing both CD3 and CD28 pathways in vitro can expand cells in a unique manner based on how both signals are presented to human T cells and the cytokines that are provided. Studies have now shown that different expansion methods and the modification of such methods can lead to T cells displaying more desirable phenotypes (15–17). Parameters such as ligand density (18), mechanical forces (19, 20), strength of the interaction (21), and duration of the signal that activates human T cells affect the outcome of the ACT response (22). Triggering T cell activation through different formats of signal delivery is therefore expected to skew the final T cell phenotype.

In this report, we have engineered a highly potent, monomeric protein that expands human T cells ex vivo when added as a soluble factor at low concentrations. The potency of this T cell expansion protein (termed T-CEP) was compared with an established protocol where an immobilized agonistic anti-CD3 mAb is combined with a soluble anti-CD28 mAb. T-CEP was also compared with a commercially available soluble T cell activator that targets both human CD3 and CD28 in the format of a combined solution of monospecific tetrameric antibody complexes (TACs). T-CEP was shown to engage both CD3 and CD28 on human T cells. The concomitant triggering of both signaling pathways led to a high level of T cell proliferative activity. Furthermore, T-CEP demonstrated an improved ability to expand CD8^+^ human T cell populations, exhibiting a less differentiated T cell phenotype that is clinically linked to more successful ACT outcomes in patients.

## Results

### Design and production of T-CEP

**Figure 1:**
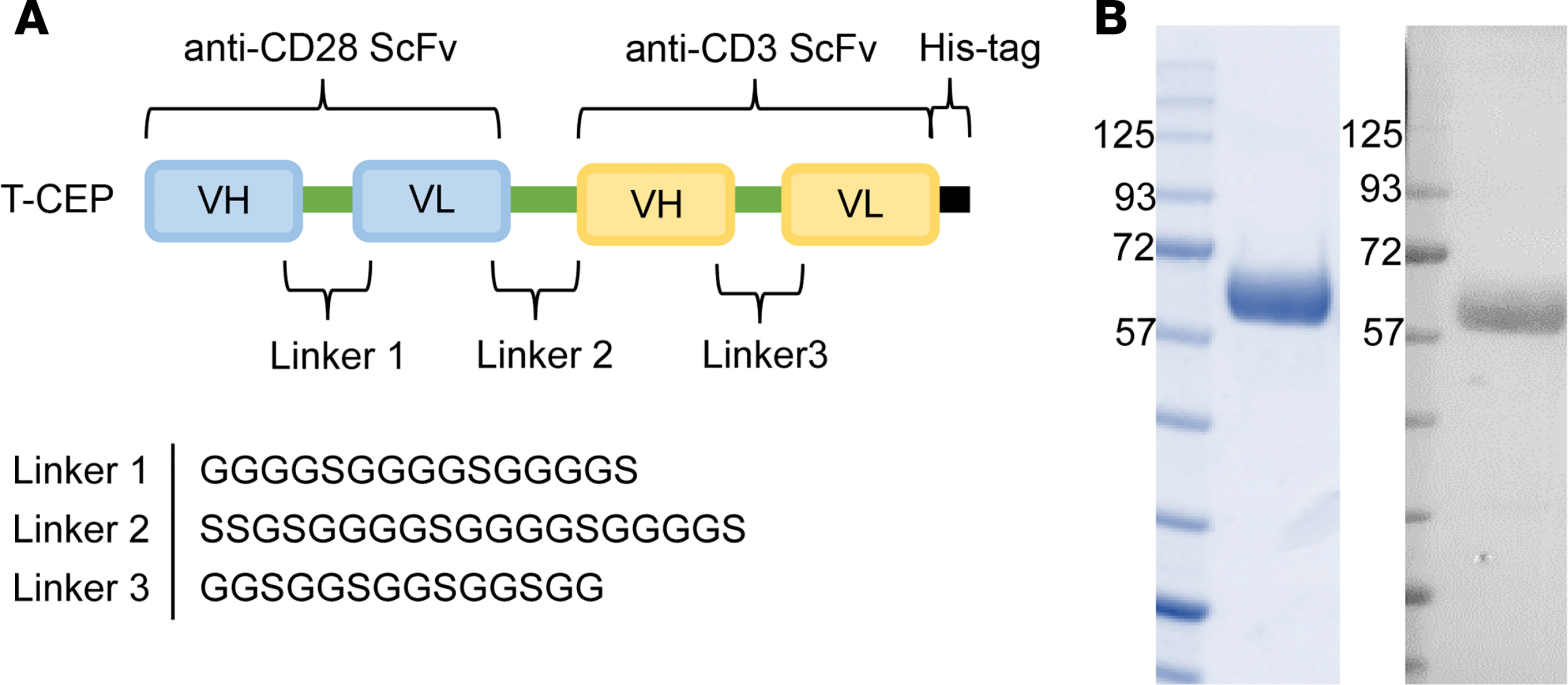
Design and purification of T-CEP. (**A**) Diagram depicting the structure of T-CEP, a T cell expansion protein. T-CEP is a bispecific agent composed of an N-terminal agonistic CD28-targeting scFv connected by a short flexible linker (Linker 2) to an agonistic CD3-binding scFv. Both scFvs are further defined as linear assemblies of variable heavy (VH) and variable light (VL) chains. A histidine tag (6x His) was inserted at the C-terminus of T-CEP for purification and detection purposes. (**B**) Recombinant T-CEP was produced in Expi293F cells and purified by Ni-NTA affinity chromatography. Its purity was confirmed by SDS-PAGE (left panel; Coomassie staining) and by Western blot (right panel; detected using an anti-His tag antibody). The protein migrates as an approximately 60 kDa band.

A 60 kDa soluble T-CEP was designed by fusing 2 distinct single chain variable fragments (scFvs) that respectively bind to human CD3 and human CD28 and act as agonists in activating both signaling pathways (Figure 1A). These selected scFv sequences have independently been shown to agonize CD3 and CD28, respectively, and accordingly activate human T cells. These scFvs were linked using a short flexible spacer (SSGSGGGGSGGGGSGGGGS), similar to previously described linkers (23). A 6-histidine tag was added at the C-terminus of the construct for purification purposes and for detecting the construct (Figure 1A). Of note, 2 T-CEP constructs were originally built to assess whether the orientation (i.e., the anti-CD28 scFv at the C-terminus or the N-terminus of the construct) had an impact on their functional properties. The construct as depicted in Figure 1A, with the scFv targeting CD28 at the N-terminus, and the CD3 scFv placed at its C-terminus, was ultimately chosen as the lead compound, based on higher expression yields and being more potent in terms of expanding T cells (results not shown). T-CEP was expressed as a secreted, soluble protein produced in Expi293F cells (Expi293 expression system) and purified using immobilized metal affinity chromatography. Its purity was confirmed by SDS-PAGE and Western blot analysis, migrating as a 60 kDa band, corresponding to the expected molecular weight of T-CEP based on its amino acid sequence (Figure 1B).

Based on its structure, T-CEP is an agent that is designed to engage signals for TCR activation and costimulation at a molar ratio of 1:1. The other currently available commercial soluble T cell activator (STEMCELL Technologies) delivers these signals in the form of soluble, monospecific TACs, complexes of agonistic mAbs against CD3 or CD28 noncovalently associated together through their Fc domains (24). The prepared TACs are then combined in one solution at a 1:1 ratio of anti-CD3 TACs and anti-CD28 TACs, resulting in an expected component ratio of 1:1.

### T-CEP binds to the human CD3 and CD28 extracellular domains

**Figure 2:**
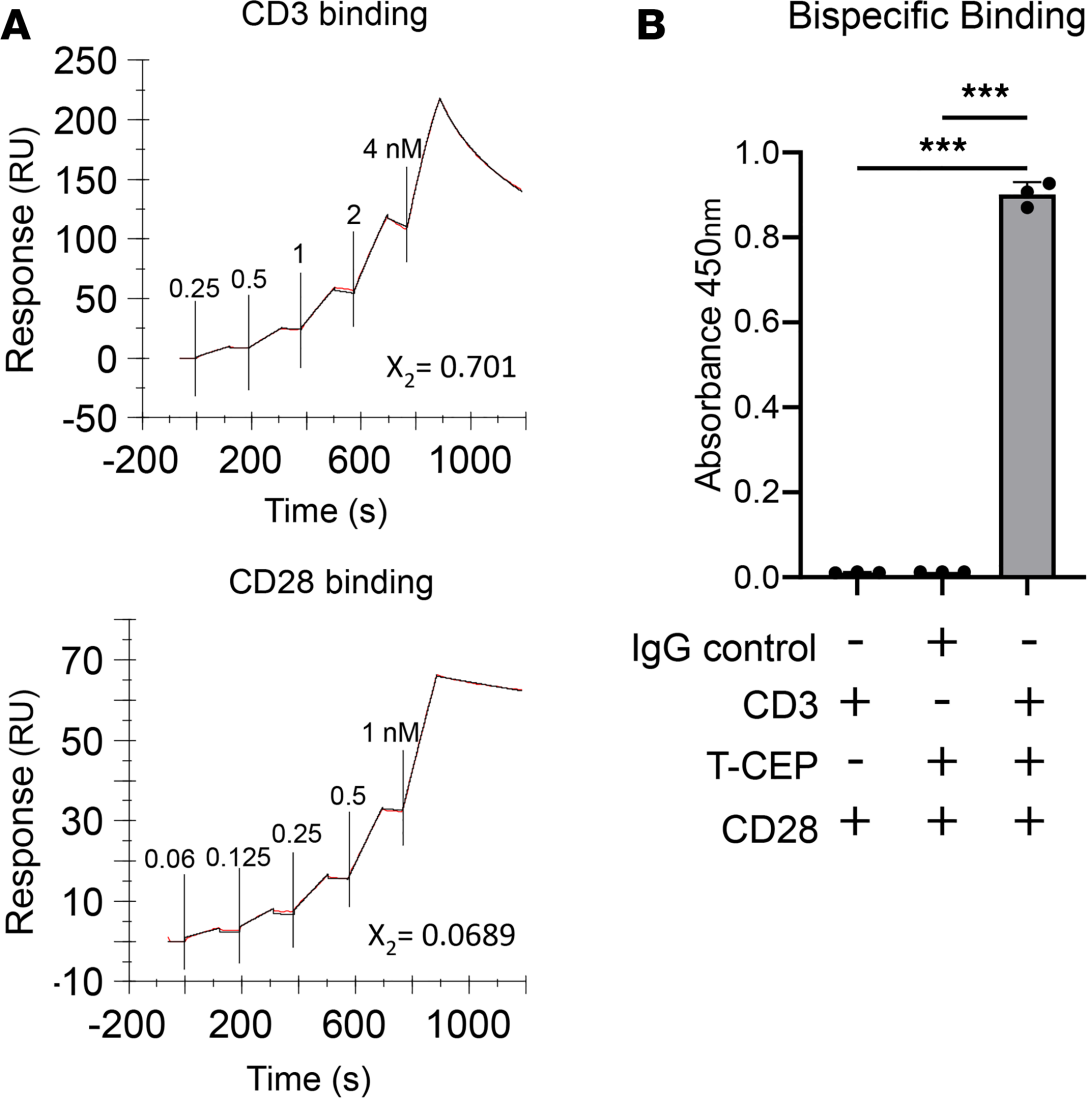
Characterization of T-CEP binding to human CD3 and human CD28 extracellular domains. (**A**) Surface plasmon resonance (SPR) sensorgrams depicting the binding of T-CEP to recombinant human Fc-tagged CD3εδ and recombinant human Fc-tagged CD28 over a range of T-CEP concentrations to calculate kinetic parameters using the 1:1 binding model using Biacore evaluation software. (**B**) Enzyme-linked immunosorbent assay (ELISA) confirming the binding of soluble T-CEP to plate-bound recombinant human CD3-Fc or an IgG control. The CD3-bound T-CEP was subsequently detected using a biotinylated recombinant human CD28-Fc construct followed by a streptavidin-HRP conjugate (*n* = 3, 1-way ANOVA with a Tukey’s multiple-comparison test). ***P < 0.001.

The binding of T-CEP to the extracellular domains of CD3 and CD28 was confirmed by surface plasmon resonance (SPR). Specifically, Fc-tagged recombinant human CD28 and human CD3Ɛδ proteins were immobilized on a protein G–modified chip, and increasing concentrations of T-CEP were flown over each immobilized target. Resulting sensorgrams were analyzed (Biacore T200 evaluation software) to calculate binding kinetic parameters (fitted to a Langmuir 1:1 binding model, Figure 2A and Table 1). T-CEP was shown to bind tightly to both CD3 and CD28 targets with equilibrium *K_D_*s of 0.48 nM and 0.24 nM, respectively. The dissociation rate (k_off_) of T-CEP binding to CD3 was 4.3 × 10^–2^/s, 2 orders of magnitude greater than the dissociation of T-CEP binding to CD28. T-CEP binding to CD3Ɛδ had a lower *K_D_* (higher affinity), higher k_on_, and faster dissociation rate relative to anti-CD3 mAbs binding to CD3Ɛδ (25). A relatively fast k_off_ represents a key factor in efficiently activating T cells, as it contributes to the turnover of TCR-MHC-peptide interactions (26).

Binding to both targets simultaneously was analyzed using an enzyme-linked immunosorbent assay (ELISA). Consistent with the SPR results, both recombinant proteins bound T-CEP alone as evident by a positive signal for Fc-tagged recombinant CD3Ɛδ and CD28 individually. In contrast, no signal was observed in the case of an Fc-matched control (Supplemental Figure 1; supplemental material available online with this article; https://doi.org/10.1172/jci.insight.141293DS1). To determine whether T-CEP could bind both targets simultaneously, T-CEP was first captured using immobilized recombinant CD3Ɛδ, then detected using recombinant CD28. CD3Ɛδ-Fc or the Fc control was first coated onto an ELISA plate and T-CEP subsequently added to wells, followed by incubation with biotinylated human CD28-Fc extracellular domain and detection with streptavidin-HRP. A positive signal was observed only in wells that contained both CD3Ɛδ-Fc and T-CEP, indicating that T-CEP is able to bind both targets at once. In contrast, wells coated with an Fc control or lacking T-CEP did not bind biotinylated CD28-Fc (Figure 2B).

### Functional characterization of T-CEP on human T cells

#### T-CEP induces the proliferation of both human CD4^+^ and CD8^+^ T cells at very low concentrations.

**Figure 3:**
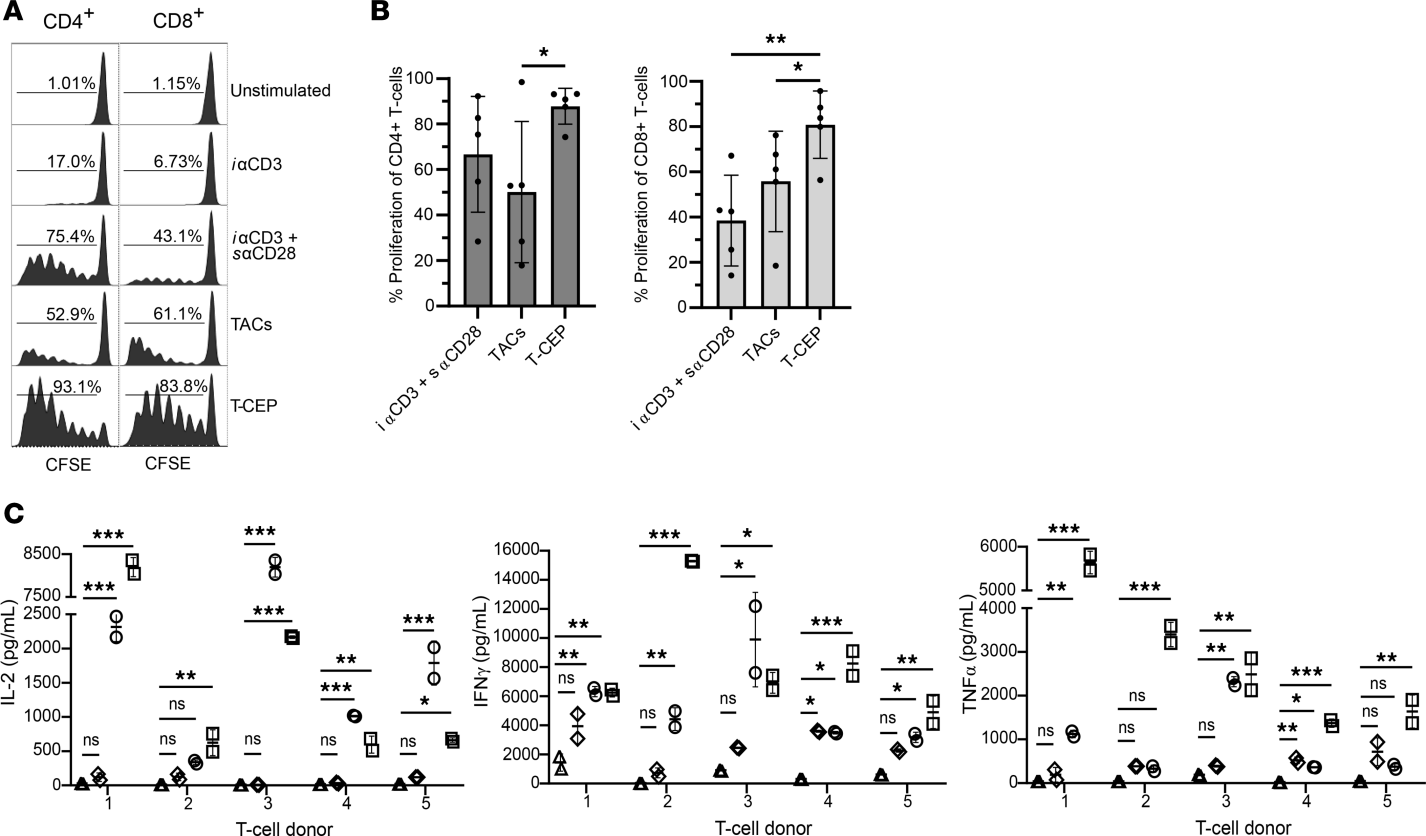
Low concentrations of T-CEP promote the ex vivo activation and proliferation of human T cells. (**A**) Representative CFSE profiles depicting the proliferation status of human T cells after a 5-day exposure to T cell–expanding conditions, without added cytokines. Immobilized anti-CD3 (i αCD3) and soluble αCD28 (s αCD28) agonistic mAbs were used at working concentrations of 5 μg/mL and 1 μg/mL, respectively, while the final concentration of TACs in wells was approximately 1.5 μg/mL. T-CEP was dispensed into wells to a final concentration of 10 ng/mL (170 fM). Similar T cell expansion profiles were observed from PBMCs isolated from 5 donors. (**B**) The early proliferative capabilities of T-CEP as measured by CFSE (day 5 after stimulation). T-CEP stimulation led to consistently elevated proliferation levels of CD4^+^ and CD8^+^ T cells, where both CD4^+^ and CD8^+^ T cell proliferation levels were significantly higher than recorded for cells treated with TACs or i αCD3 with s αCD28 (*n* = 5 donors, 1-way repeated measures ANOVA with a Tukey’s multiple-comparison test). (**C**) Representative cytokine secretion levels observed at day 5 for human T cells exposed ex vivo to cell expansion conditions. Cytokine secretion profiles observed for each of 5 T cell donors (*n* = 2, 1-way ANOVA with Dunnett’s test). TACs, tetrameric antibody complexes; i αCD3, immobilized anti-CD3; s αCD28, soluble anti-CD28. *P < 0.05; **P < 0.01; ☐, T-CEP; ○, TACs; ◇, i αCD3 + s αCD28; △, i αCD3.

To determine the optimal working concentration of T-CEP, T cells isolated from human PBMCs were stained with carboxyfluorescein succinimidyl ester (CFSE) (a cell proliferation tracer) and stimulated with T-CEP for 5 days over a range of T-CEP concentrations (50 pg/mL up to 10 μg/mL). Their proliferative activity, as evidenced by a loss in CFSE signal, was analyzed by flow cytometry (results not shown). Comparable T cell proliferative activity by T-CEP was noted for concentrations as low as 500 pg/mL. However, for all subsequent studies, a T-CEP concentration of 10 ng/mL (170 fM) was selected as it consistently yielded maximal T cell proliferation (Figure 3A). Interestingly, the concentration of soluble T-CEP required to cause a full expansion of human T cells ex vivo was 150-fold less (in g/L) than required for TACs (when used per manufacturer instructions, ~1.5 μg/mL, ~2.5 pM). This T-CEP concentration was also far less than the optimal doses of i αCD3 (5 μg/mL) and s αCD28 (1 μg/mL) mAbs. As expected, unstimulated T cells did not proliferate while anti-CD3 without costimulation led to a much-reduced level of both CD4^+^ and CD8^+^ human T cell proliferation. Over 5 donors, significantly greater proliferation was induced after 5 days using T-CEP relative to TACs in CD4^+^ T cells (*P* < 0.05), and CD8^+^ T cells (*P* < 0.05), demonstrating that on average 87.8% of the CD4^+^ and 80.9% of the CD8^+^ T cells had undergone proliferation according to CFSE levels (Figure 3B).

Cytokines released into the culture medium by activated human CD3^+^ T cells were measured at day 5, as shown by cytokine secretion profiles (Figure 3C). Both T-CEP (10 ng/mL) and TACs (~1.5 μg/mL) induced the production of IL-2 at higher levels than when T cells were stimulated with i αCD3 and i αCD3 with s αCD28 mAbs. T-CEP also showed high levels of IFN-γ and TNF-α secretion. Across all 5 donors, IL-2, IFN-γ, and TNF-α secretion by T-CEP was significantly increased relative to i αCD3, yielding the highest level of TNF-α secretion in each donor. The high secretion of cytokines 5 days after stimulation relative to the other methods demonstrates the superior ability of T-CEP to induce early activation and proliferation at fM concentrations.

#### T-CEP leads to the activation and expansion of T cells over the course of 12 days.

**Figure 4:**
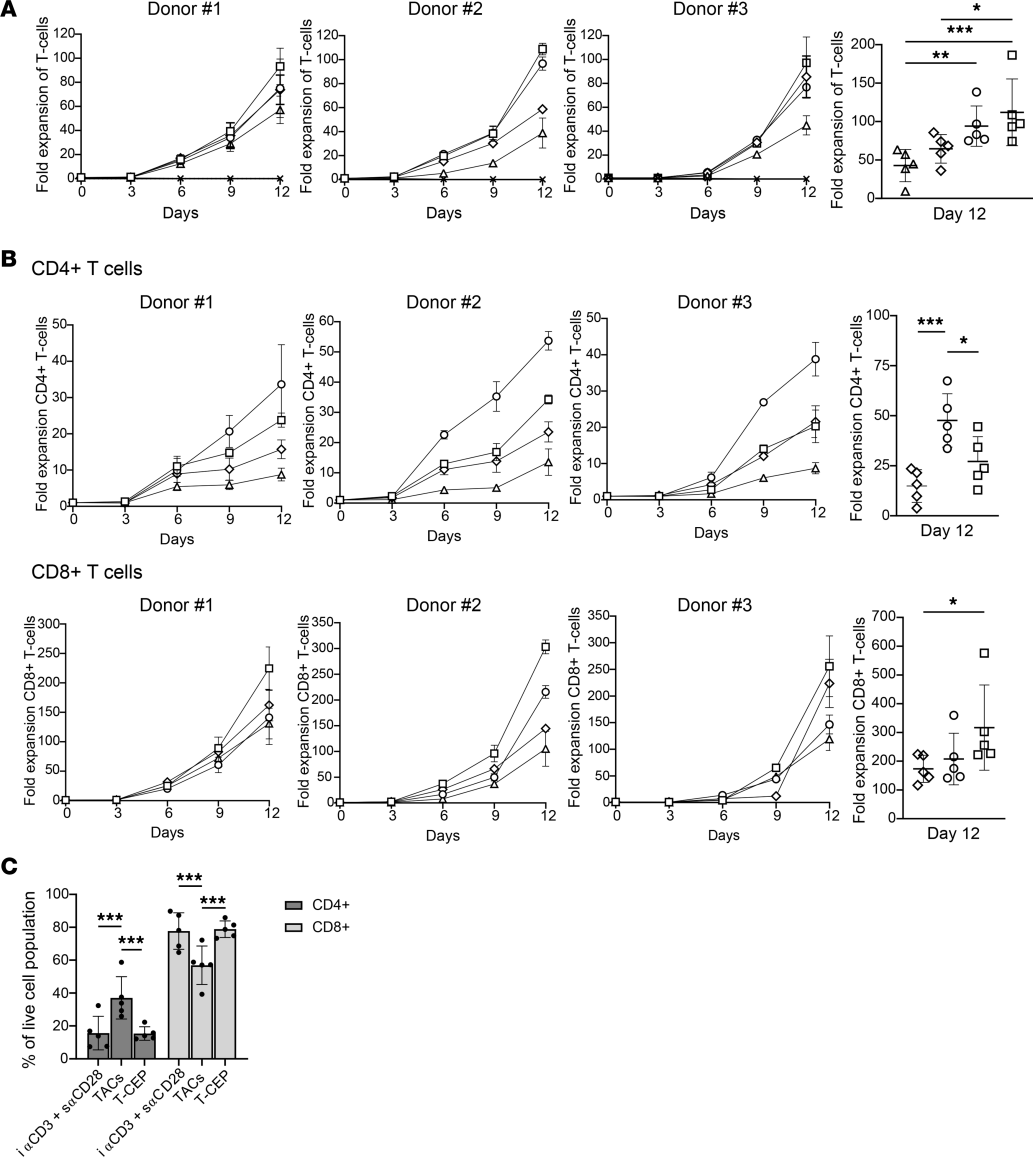
Monitoring the ex vivo expansion of activated human T cells over 12 days. (**A**) 12-Day expansion of purified human T cells from 5 PBMC donors, with representative expansion profiles of 3 donors. For each of the 5 donors, the number of viable cells was counted in duplicate samples using a hemocytometer following the staining of dead cells with trypan blue for each expansion method tested (right side, scatter plot). The average levels of T cell expansion were statistically greatest when T cells were treated with T-CEP, as compared with i αCD3, i αCD3 + s αCD28, and TACs. (*n* = 5, 1-way repeated measures ANOVA, with a Tukey’s multiple-comparison test.) (**B**) 12-Day expansion of CD4^+^ and CD8^+^ T cell subsets from purified T cells of 5 PBMC donors, along with individual expansion profiles of 3 representative donors. Scatter plots (right side) illustrate the effect of each treatment on the observed fold changes in the expansion of CD4^+^ or CD8^+^ T cells for each of 5 donors. The fold expansion was calculated based on the starting number of CD4^+^ or CD8^+^ T cells. T-CEP treatment resulted in the highest fold expansion of CD8^+^ T cells (*n* = 5, 1-way repeated measures ANOVA with a Tukey’s multiple-comparison test). (**C**) The percentage of activated human CD4^+^ and CD8^+^ T cell populations relative to the total number of viable lymphocytes as defined by flow cytometry following 12 days of culture. T-CEP or the combination of immobilized i αCD3 and soluble s αCD28 mAbs favored the expansion of CD8^+^ human T cells significantly more than TACs. (*n* = 5, 1-way repeated measures ANOVA with a Tukey’s multiple-comparison test.) TACs, tetrameric antibody complexes; i αCD3, immobilized anti-CD3; s αCD28, Soluble anti-CD28. *P < 0.05; **P < 0.01; ***P < 0.001; ☐, T-CEP; ○, TACs; ◇, i αCD3 + s αCD28; △, i αCD3; x, unstimulated.

T cells used for ACT are required to undergo a rapid expansion phase lasting 12–14 days before reinfusion into the patient (1). Human CD3^+^ T cells isolated from PBMCs of 5 healthy donors were expanded over the course of 12 days after a single stimulation, with IL-2 being added to the medium to further aid in cell expansion and activation ex vivo. Cells were transferred to G-Rex plates 3 days into the 12-day expansion stage. Every subsequent 3 days, aliquots were taken from duplicate wells to record the number of viable T cells present. T cells from the 5 donors were stimulated with T-CEP (10 ng/mL; 170 fM) or soluble TACs (25 μL/mL, ~1.5 μg/mL) or with i αCD3 with or without s αCD28 (Figure 4A). The highest levels of total T cell expansion were observed on day 12, when T cells were treated ex vivo with soluble expansion methods. Importantly, T-CEP accounted for the highest T cell expansion in 4 of 5 PBMC donors (Supplemental Figure 2). As expected, unstimulated T cells did not expand, while T cells treated with i αCD3 alone displayed a reduced level of T cell expansion relative to methods that included CD28 costimulation. All these approaches, however, yielded lower average levels of expansion than by T-CEP, with significant increases observed for T-CEP relative to immobilized methods (*P* < 0.05) (Figure 4A).

T cells were further analyzed by flow cytometry to monitor the expansion of CD4^+^ and CD8^+^ T cell subsets over 12 days for the 5 donors, relative to the starting number of CD4^+^ or CD8^+^ T cells before stimulation (Figure 4B). Treatment with TAC induced the highest level of human CD4^+^ T cell expansion after 12 days (mean: 48-fold, range: 34- to 67-fold). This increase was significantly greater than observed for i αCD3 alone (mean: 8-fold, range: 1- to 13-fold) (*P* < 0.0001), i αCD3 with s αCD28 (mean: 15-fold, range: 4- to 20-fold) (*P* < 0.001), and T-CEP (mean: 27-fold, range: 13- to 45-fold) (*P* < 0.05). In contrast, T-CEP favored the expansion of CD8^+^ T cells, with the highest CD8^+^ T cell expansion in each of the 5 PBMC donors, resulting in a 317-fold mean expansion rate (range: 222-to 576-fold). This level of expansion was significantly greater than with i αCD3 alone (mean: 119-fold, range: 30- to 208-fold) (*P* < 0.005) or after stimulation with i αCD3 with s αCD28 (mean: 173-fold; range, 116- to 224-fold) (*P* < 0.05), while also trending toward significance compared with stimulation with TACs (*P* = 0.06) (mean: 208-fold, range: 141- to 360-fold).

To further assess the extent of CD4^+^ and CD8^+^ T cell subsets’ expansion after 12 days under each expansion condition, we determined by flow cytometry the percentages of CD4^+^ and CD8^+^ T cell populations relative to the total population of viable lymphocytes recovered from the 5 donors (Figure 4C). Comparing T-CEP with other conditions that provide costimulation indicated that T-CEP favored the expansion of CD8^+^ T cells over CD4^+^ T cells, and a similar ratio of T cells was produced when treated with a conventional method of i αCD3 with s αCD28. In contrast, T cell treatment with TACs led to a significantly reduced percentage of CD8^+^ T cells (*P* < 0.0001) and an increased percentage of CD4^+^ T cells (*P* < 0.0001). Before stimulation, the CD4^+^/CD8^+^ ratios of T cells purified from healthy donor PBMCs were between 2.1 and 2.7. After stimulation with T-CEP, the CD4^+^/CD8^+^ ratio ranged from 0.15 to 0.3.

**Figure 5:**
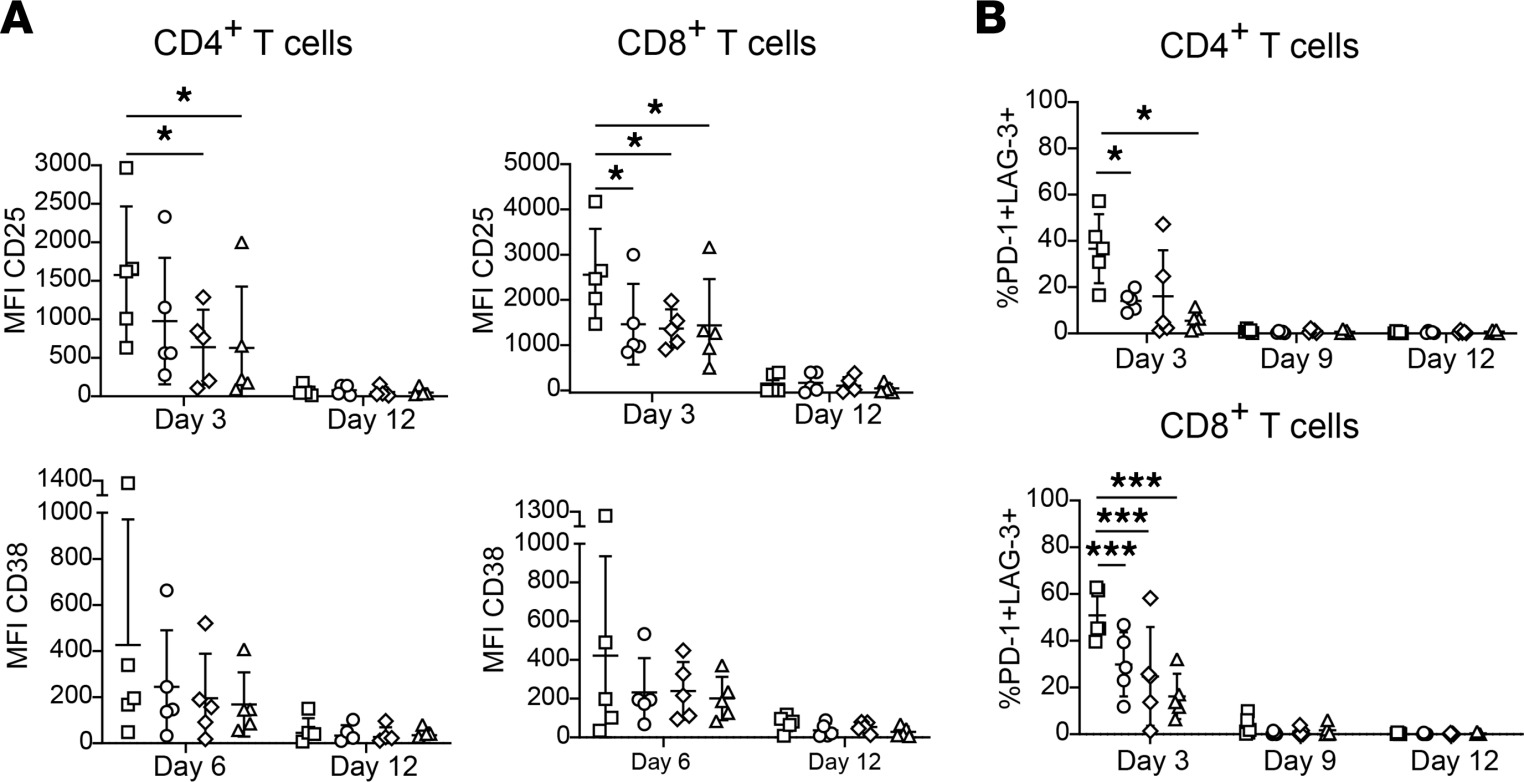
Human T cell activation following stimulation over a 12-day ex vivo expansion period. The presence of surface markers on human T cells from 5 donors was analyzed by flow cytometry. (**A**) The surface expression of activation markers CD25 and CD38 exposed to specific stimulation conditions. The increase in median fluorescence intensities (MFIs) for the activation markers was calculated by subtracting MFI values at day 0 before stimulation. MFI values of both activation markers decreased to baseline values by day 12 for all 3 donors. T-CEP–stimulated T cells showed the highest average expression of both activation markers. The MFI of CD25, significantly increased when stimulated with T-CEP as compared with i αCD3 and i αCD3 with s αCD28 in CD4^+^ T cells, and compared with i αCD3 in CD8^+^ T cells. There was no significant difference in peak CD38 MFI values between specific stimulation methods. By day 12 the MFI of both activation markers returned to pretreatment levels in all stimulation methods (*n* = 5, 1-way repeated measures ANOVA with a Tukey’s multiple-comparison test). (**B**) The coexpression of PD-1 and LAG-3 markers on human T cells peaked by day 3 and returned to pretreatment levels by day 12. In both CD4^+^ and CD8^+^ T cells, the expression of these markers was significantly increased on day 3 in T-CEP–stimulated cells but returned to baseline values as in the case of other activation methods by day 9. (*n* = 5, 1-way repeated measures ANOVA with a Tukey’s multiple-comparison test.) TACs, tetrameric antibody complexes; i αCD3, immobilized anti-CD3; s αCD28, soluble anti-CD28. *P < 0.05; ***P < 0.001; ☐, T-CEP; ○, TACs; ◇, i αCD3 + s αCD28; △, i αCD3.

The levels of activation markers CD25 and CD38 on human CD4^+^ and CD8^+^ T cells from the 5 donors were monitored by flow cytometry at specific time points during the 12-day expansion period (Figure 5A). The increase in median fluorescence intensity (MFI) of CD25 expression peaked on day 3 and on day 6 for CD38. T-CEP induced the highest average level of activation markers on the peak day in the CD8^+^ and CD4^+^ T cell populations. Specifically, the observed CD25 MFI values on day 3 were highest in the T-CEP–treated CD4^+^ T cell population (mean MFI 1577), which was significant relative to the same population of T cells expanded with either i αCD3 with s αCD28 (MFI 640, *P* < 0.05) or i αCD3 (MFI 629, *P* < 0.05), though not significant when compared with TACs (MFI 1463). Again, the average CD25 MFI values on day 3 were highest in the T-CEP–treated CD8^+^ population (mean MFI 2558), although only significantly higher than i αCD3 with s αCD28 (MFI 1368, *P* < 0.05), not i αCD3 (MFI 1427) alone or TACs (MFI 1463) treated CD8^+^ cell populations. In both CD4^+^ and CD8^+^ T cells, the overall trend in CD38 expression showed highest CD38 MFI values on day 6, with the highest average values in the 5 donors being observed after T-CEP stimulation. However, this trend was not statistically significant. On day 12 the levels of both activation markers were comparable to that before stimulation, regardless of the expansion method used or the activation marker being monitored.

The surface expression of exhaustion markers programmed cell death protein 1 (PD-1) and lymphocyte activation gene 3 (LAG-3) coexpressed on CD4^+^ and CD8^+^ T cells was analyzed by flow cytometry for each expansion condition (Figure 5B). The percentage of CD4^+^ and CD8^+^ T cells coexpressing these markers peaked on day 3 for all expansion methods tested. In 5 donors, the day 3 coexpression was significantly higher in CD4^+^ and CD8^+^ T cells stimulated with T-CEP in both CD4^+^ T cells (mean: 36.6%) and CD8^+^ T cells (50.9%) as compared with all the other tested expansion methods (CD4^+^ T cells: i αCD3 5.5%, i αCD3 with s αCD28 16.1%, TACs 14.0%; CD8^+^ T cells: i αCD3 16.2%, i αCD3 with s αCD28 24.7%, TACs 29.9%). However, this expression pattern returned to preactivation levels by day 9 and day 12 following each stimulation method.

### Stimulation with T-CEP leads to distinct human T cell phenotypic differentiation

**Figure 6:**
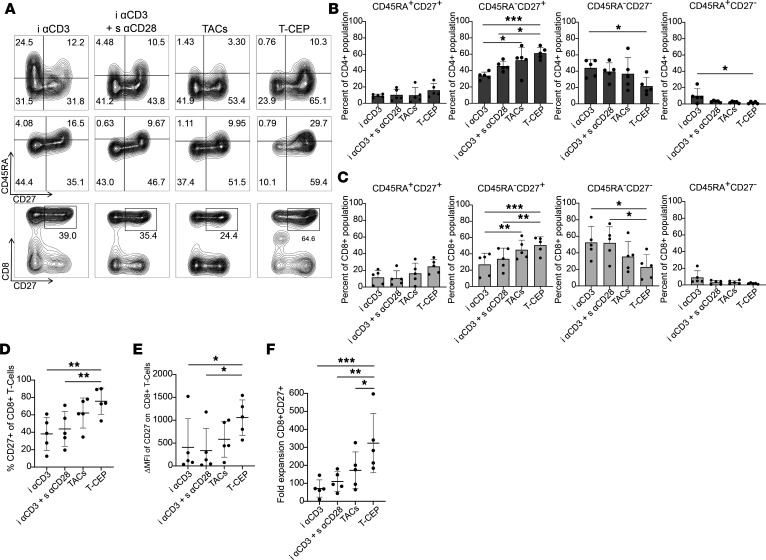
T-CEP favors the ex vivo differentiation of T cells toward a less differentiated human CD8^+^ T cell phenotype. (**A**) Representative cytometric plots highlighting the expression of CD45RA and CD27 on CD4^+^ (top row) and CD8^+^ (bottom row) T cells and of CD8^+^CD27^+^ human T cells at day 12 following their activation and expansion using a designated treatment. (**B**) Surface expression at day 12 of CD45RA and CD27 markers as a percentage of CD4^+^ populations from 5 donors. In the CD3^+^CD4^+^ T cell subset population, T-CEP displayed significantly increased levels of CD45RA^–^CD27^+^ T cells relative to i αCD3 treated cells with or without s αCD28. Additionally, T-CEP–stimulated T cells had the lowest percentage of CD45RA^–^CD27^–^ and CD45RA^+^CD27^–^ phenotype, which were significantly reduced compared with i αCD3–treated T cells (*n* = 5, 1-way repeated measures ANOVA with a Tukey’s multiple-comparison test). (**C**) Surface expression at day 12 of CD45RA and CD27 markers as a percentage of CD8^+^ populations from 5 donors. On average, T-CEP–stimulated T cells displayed the highest percentage of CD45RA^–^CD27^+^ phenotype, with a significant increase relative to i αCD3 and i αCD3 with s αCD28. T-CEP also significantly reduced the CD45RA^–^CD27^–^ T cell phenotype relative to i αCD3 with or without s αCD28 (*n* = 5, 1-way repeated measures ANOVA with a Tukey’s multiple-comparison test). (**D**) The percentage of viable CD8^+^CD27^+^ lymphocytes at day 12 observed in human T cell samples collected from 5 donors. T-CEP–stimulated T cells coexpressing CD8 and CD27 accounted for 62.3% of the total viable lymphocyte population, which was significantly higher compared with cells stimulated with i αCD3 alone, i αCD3 and s αCD28, and TACs (*n* = 5, 1-way repeated measures ANOVA with a Tukey’s multiple-comparison test). (**E**) CD27 expression on CD8^+^ T cells (ΔMFI) at day 12 from each treatment modality. The ΔMFI was calculated by subtracting the MFI value of the appropriate isotype control. The subset of CD8^+^ T cells expressing CD27 was significantly higher in T cells stimulated with T-CEP relative to i αCD3 or i αCD3 and s αCD28 (*n* = 5, 1-way repeated measures ANOVA). (**F**) The expansion of the CD8^+^CD27^+^ T cells over 12 days was substantially greater when treated with T-CEP than with the other indicated stimulation methods (*n* = 5, 1-way repeated measures ANOVA with a Tukey’s multiple-comparison test). TACs, tetrameric antibody complexes; i αCD3, immobilized anti-CD3; s αCD28, soluble anti-CD28. *P < 0.05; **P < 0.01; ***P < 0.001.

The differentiation patterns of human T cells from 5 donors were analyzed for the 12-day period. In particular, the expression levels of CD45RA and CD27 were monitored on CD4^+^ and CD8^+^ cells to provide an estimate of the differentiation state of these T cell subsets (Figure 6A). Specifically, CD27 plays a role in T cell activation and differentiation, and its expression has been associated with in vivo persistence of T cells (2, 27), while the loss of CD27 expression generally indicates late or terminal differentiation (28). Meanwhile, CD45RA is expressed on early Tn and Tscm cell subsets, and its expression is lost upon differentiation into Tcm and Tem cells. This marker is then reexpressed upon differentiation into a late-stage Temra subset (29, 30). Therefore, it is expected that T cell subsets would display the following expression pattern of these 2 markers as follows: CD45RA^+^CD27^+^ < CD45RA^–^CD27^+^ < CD45RA^–^CD27^–^ < CD45RA^+^CD27^–^ going from least to furthest differentiated states. After 12 days of expansion ex vivo, the T-CEP–treated human CD4^+^ T cell population in each donor exhibited relatively low percentages of the more differentiated populations (Figure 6B). Over the 5 donors, the average percentage of CD45RA^+^CD27^–^ cells in the CD4^+^ population was the lowest after T-CEP expansion (1.5%), while the CD45RA^–^CD27^–^ population was also lower than all other tested methods (21.6%). T-CEP was the only method to significantly reduce the average CD45RA^–^CD27^–^ population compared with i αCD3 alone (44.0%) (*P* < 0.05). Meanwhile, the CD45RA^–^CD27^+^ phenotype in the CD4^+^ T cell population was significantly increased when treated with T-CEP as compared with T cells treated with i αCD3 alone (*P* < 0.001) and i αCD3 with s CD28 (*P* < 0.05). This increase in CD45RA^–^CD27^+^ phenotype was also seen following treatment with TACs but to a lesser extent (Figure 6B). Similar phenotypic changes were observed in the T-CEP–treated CD8^+^ T cell population after 12 days, where the more differentiated CD45RA^+^CD27^–^ T cells made up on average 1.8% of the population in the 5 donors, and the CD45RA^–^CD27^–^ T cell population accounted on average for 22.8% of total CD8^+^ T cells (Figure 6C). In comparison, treatment with TACs led to an average of 36.1% of CD8^+^ T cells having a CD45RA^–^CD27^–^ phenotype. Out of these 2 soluble activators, only T-CEP significantly reduced this phenotype in the CD8^+^ T cell population as compared with i αCD3 with s αCD28 (52.0%, *P* < 0.05) or i αCD3 alone (52.5%, *P* < 0.05). For the T-CEP–stimulated T cells, this reduction in the CD45RA^–^CD27^–^ phenotype appeared to shift more toward CD27^+^ phenotypes, as the less differentiated CD45^+^CD27^+^ and CD45RA^–^CD27^+^ populations were highest in T-CEP–treated T cells, with a mean population percentage of 24.6% and 50.7%, respectively (Figure 6C). This percentage of CD45RA^–^CD27^+^ T cell subset was significantly increased as compared with cells stimulated with i αCD3 alone (26.9%, *P* < 0.001) or i αCD3 with s αCD28 (33.8%, *P* < 0.01). As seen with the CD4^+^ population, the TAC-treated samples also had increased levels of CD45RA^–^CD27^+^ T cells, but again to a lesser extent than observed with T-CEP (Figure 6C).

The number of transferred CD8^+^CD27^+^ T cells has previously been associated with an improved patient response to the adoptive therapy treatment using autologous TILs for metastatic melanoma (2). Stimulation with T-CEP increased the number of CD8^+^CD27^+^ T cells relative to the other methods of stimulation for each of the 5 donors (Figure 6D), with a mean frequency of CD8^+^CD27^+^ cells representing 62.3% of the viable lymphocyte population on day 12. This percentage was significantly greater than the proportion of such cells observed under other expansion conditions (i αCD3, 34.3%, *P* < 0.01; i αCD3 and s αCD28, 37.3%, *P* < 0.01; TACs, 38.1%, *P* < 0.01). This elevated population of CD8^+^CD27^+^ T cells upon T cell expansion with T-CEP was independent of the T-CEP concentration over a 10 ng/mL to 10 μg/mL range (54%–78% of total viable lymphocyte population), which contrasted with TACs (range of 23%–57%) (data not shown). Higher expression of CD27 was consistently observed for CD8^+^ T cells in the T-CEP–treated group in each donor on day 12, as demonstrated by the greater change in median fluorescence intensity (ΔMFI) of CD27 (mean ΔMFI 1057) relative to T cell groups treated with either i αCD3 (mean ΔMFI 407, *P* < 0.05) or i αCD3 and s αCD28 (mean ΔMFI 336, *P* < 0.05). This elevated CD27 expression compared with the immobilized methods was also observed in stimulation through TACs but with an average ΔMFI of CD27, about half of what was observed through T-CEP stimulation (mean ΔMFI 583) (Figure 6E). The fold expansion of CD8^+^CD27^+^ T cells by day 12 for each donor (*n* = 5) was calculated using the percentage of CD8^+^CD27^+^ cells derived from viable cells and the average total T cell count, relative to each donor initial CD8^+^CD27^+^ T cell count. On average, T-CEP was found to increase the CD8^+^CD27^+^ T cell count by 325-fold (range: 184–603) over 12 days, with the highest fold expansion in each donor. In comparison, i αCD3 (71-fold, range: 11–150, *P* < 0.001), i αCD3 and s αCD28 (110-fold, range: 44–185, *P* < 0.01), or TACs (172-fold, range: 65–330, *P* = 0.05) had a significantly lower amount after the 12-day expansion as determined for the 5 donors (Figure 6F).

## Discussion

The ex vivo expansion of T cells through CD3 and CD28 pathways’ coactivation is a common strategy for generating sufficient T cell products needed for ACT, although improvements to existing methods that would favor the expansion of fully functional CD8^+^ T cells are still needed. Existing protocols make use of immobilized CD3 and CD28 agonistic antibodies or ligands, presented typically on cells or beads. It has been reported that common ACT approaches where anti-CD3 and anti-CD28 are immobilized on magnetic beads favor the expansion of human CD4^+^ T cell subsets (16), which typically require sustained antigen exposure for growth (31, 32). Longer expansion times eventually favor the expansion of preferred human CD8^+^ T cells (33). However, methods that enrich for CD8^+^ T cells before expansion are clinically preferred because of the critical antitumor activity of CD8^+^ T cells (34). More recently, monospecific TACs have been employed as soluble T cell activators. However, we show here that TACs provide a reduced ability to expand CD8^+^ T cells that are clinically needed for successful ACTs.

We designed a simple soluble factor, termed T-CEP, that simultaneously delivers agonistic signals to both CD3 and CD28 on human T cells (Figure 1 and Figure 2). Structurally, T-CEP is a bispecific protein composed of a linear arrangement of 2 scFv domains that agonize CD3 and CD28, respectively. The use of T-CEP results in the enhanced expansion of nonterminally differentiated human T cells that contrast with existing CD3/CD28 activation methods (Figure 3, Figure 4, Figure 5, and Figure 6). Specifically, T-CEP binds to recombinant human CD3 and CD28 with low nanomolar binding constants (Table 1) and as a soluble factor can activate both human CD4^+^ and CD8^+^ T cells at concentrations in the fM range (10 ng/mL or less) relative to other known soluble CD3/CD28 agents (typically μg/mL range; Figure 3). Human T cells stimulated with T-CEP and cultured for 5 days, without additional added cytokines, are able to induce their proliferation and cytokine secretion at levels similar to or higher than soluble TACs, but at a concentration (10 ng/mL) that is at least 150-fold less than TACs (1.5 μg/mL; Figure 3).

T-CEP demonstrates a preference for expanding CD8^+^ T cells as compared with TACs. This phenomenon is not yet understood. It has previously been shown that the transient stimulation of human T cells ex vivo favors the expansion of human CD8^+^ T cells, including when i αCD3 and anti-CD28 on beads are removed after 24 hours and cells are expanded for 14 days (17). The removal of the i αCD3 could explain in part why the expansion of human T cells may be skewed toward the production of CD8^+^ T cells after 12 days of expansion. The observed T-CEP biasing toward the expansion of human CD8^+^ T cells may reflect the degradation of T-CEP and its dilution (day 3) in the medium as its activation of human T cells requires concentrations above 1 ng/mL. It is also possible that differences in strength or duration of interactions involving CD3 and CD28 are responsible for this difference.

Obtaining less differentiated CD8^+^ T cells after expansion has been of particular interest for ACT (13), as they have demonstrated superior antitumor immunity in vivo (35, 36). Human T cells can be further characterized along their differentiation pathways, from less differentiated subsets, such as Tn, Tscm, or Tcm cells, to more differentiated phenotypes, such as Tem or Temra cell populations (13, 14). Importantly, T cell subsets at later stages of differentiation display a decline in IL-2 production and downregulate surface markers, such as CD27, CD28, CCR7, CD127, and CD62L (37, 38). Tracking such markers on T cells provides molecular signatures as to the state of differentiation of T cell populations. In particular, the surface expression of CD45RA and CD27 on human T cells collected from 5 donors was measured to estimate their state of differentiation following their ex vivo expansion with T-CEP and other activation methods. The expression of these markers on T cells has been established in the past with the least differentiated phenotypes being CD45RA^+^CD27^+^ (Tscm-like) and CD45RA^–^CCD27^+^ (Tcm-like or early Tem) and the later stage T cell subtypes being CD45RA^–^CD27^–^ (Tem) and CD45RA^+^CD27^–^ (Temra) (30, 33, 39–41). T-CEP–expanded human T cells exhibited a distinct CD27^+^ cell population in both their CD8^+^ and CD4^+^ T cell subsets (Figure 6). These CD8^+^CD27^+^ T cells were primarily Tcm-like, as CD8^+^CD45RA^–^CD27^+^ cells represented the highest percentage of cells present at day 12 (Figure 6C). Also, this level of CD8^+^CD27^+^ T cells in the final T-CEP–expanded product (62.3%) was not observed with other expansion methods (Figure 6D), with a significantly high CD8^+^CD27^+^ fold change expansion that was greatest in each of the 5 PBMC donors tested (Figure 6F). Overall, T-CEP led to a focused expansion of less differentiated Tcm-like CD8^+^CD45RA^–^CD27^+^ and Tscm-like CD8^+^CD45RA^+^CD27^+^ cells. This phenotype appeared to arise from a combination of a high level of CD27 expression along with a preferred CD8^+^ T cell expansion.

The ex vivo expansion features for T-CEP are clinically relevant, as CD8^+^ Tcm cells have previously been demonstrated to be superior to late-stage differentiated T cell subsets in ACT (35, 42). For instance, the persistence of melanoma-reactive T cells in patients with metastatic melanoma receiving ACT has been associated with their expression of CD27, suggesting a linkage between this differentiation marker and antitumor memory CD8^+^ T cells (27). Based on clinical outcomes observed in melanoma patients treated with TILs, the CD8^+^/CD4^+^ ratio and the number of CD8^+^CD27^+^ T cells infused in these patients correlated with their response to treatment (2, 43). Moreover, it was shown recently that the frequency of CD45RO^–^CD27^+^CD8^+^ T cells in the expanded CAR T cell product infused in patients with multiple myeloma was associated with their clinical response (44). CD27 is a costimulatory receptor that promotes T cell survival and memory T cell expansion (45, 46). The loss of this receptor on T cells is a marker for terminal differentiation (39, 47). T-CEP consistently favors the ex vivo expansion of CD8^+^CD27^+^ T cells after 12 days of expansion in medium supplemented with IL-2, conditions that mimic those used in ACT. A potent small recombinant protein, such as T-CEP, could provide an alternative method for expanding with minimal efforts (a single dose) a more desirable, less differentiated CD27^+^CD8^+^ T cell population as part of an ACT protocol. A rationale explaining why T-CEP potently expands human T cells at low concentrations while skewing their differentiation pattern toward mostly CD8^+^CD27^+^ T cells remains elusive. Structurally, T-CEP is a soluble, monomeric, 60 kDa, bispecific protein that engages both CD3 and CD28 through the action of 2 small scFvs linked by a short flexible spacer. As such, it may cross-link or promote the spatial rapprochement of human T cells expressing both markers. The molecular presentation of both CD3/CD28 agonists in T-CEP is also fixed at a molar ratio of 1:1. Its overall geometry differs from monospecific CD3/CD28 antibodies’ TACs, the only other known soluble T cell activator delivering these signals. Although it was not further investigated, the nature of the scFvs agonizing CD3 and CD28, respectively, may explain why T-CEP works at low concentrations leading to a distinct pattern of T cell differentiation. Combining T-CEP with cytokines, such as IL-7 and IL-15, may further skew human T cells toward less differentiated phenotypes, such as CD8^+^ Tcm (48).

From a practical perspective, T-CEP is a smaller soluble protein than TACs. It is produced well in eukaryotic cells, and less of it is required for its application in expanding human T cells ex vivo. Further investigation on the use of T-CEP in ACT appears warranted based on its ability to expand a T cell phenotype desirable for ACT.

## Methods

### Recombinant protein expression and purification.

T-CEP is a bispecific, monomeric protein construct composed of 2 agonistic scFvs that bind to human CD3 and human CD28, respectively, which are linked together through a short flexible spacer (Figure 1A). Specifically, a synthetic gene was assembled that coded for a human CD28 scFv followed by a CD3-binding scFv and a C-terminal His6-tag. The gene was synthesized to include distinct glycine-rich linkers between the VH and VL (GGGGSGGGGSGGGGS between anti-CD28 scFv VH and VL; GGSGGSGGSGGSGG between anti-CD3 scFv VH and VL) and a short spacer (SSGSGGGGSGGGGSGGGGS) linking the 2 scFv domains. The final construct was cloned into a pcDNA-3.4 expression plasmid with a 5′ Igκ leader sequence for high protein secretion in the Expi293F cells (Thermo Fisher Scientific) in the Expi-293 mammalian cell expression system (Thermo Fisher Scientific). T-CEP was purified using HisTrap HP columns (GE Healthcare) and desalted using PD-10 columns (GE Healthcare). Protein purity was assessed by SDS-PAGE and by Western blot analyses (murine anti–polyHistidine-mAb [clone His-1] peroxidase conjugate; MilliporeSigma). The concentration of the protein was quantified using A280 measurement with the extinction coefficient calculated based on sequence (ProtParam; ExPASy). Approximately 25 mg of purified T-CEP was recovered per liter of culture medium.

### Surface plasmon resonance.

The binding of T-CEP to the extracellular domains of human CD3 and CD28 was determined by SPR (Biacore T200, GE Healthcare). Briefly, protein G–coated CM5 chips (GE Healthcare) were used to capture human recombinant hIgG1-tagged CD28 (P10747-1) and hIgG1-tagged CD3Ɛδ (Cambridge Biologics). Each target protein was injected at a flow rate of 10 μL/min for 60 seconds, and the capture was monitored in real time. The binding of T-CEP to either CD3 or CD28 was measured using an increasing concentration gradient of 0.25, 0.5, 1, 2, 4 nM for T-CEP binding to recombinant CD3 and of 0.0625, 0.125, 0.25, 0.5, 1 nM for T-CEP binding to recombinant CD28. T-CEP was injected with a flow rate of 30 μL/min for 120 seconds, with a dissociation time of 300 seconds for both targets. T-CEP binding to a control lane coated with protein G without any captured target protein was also calculated and subtracted from the active lane. No binding was observed in the control lane (data not shown). The resulting curve was fitted using a 1:1 Langmuir binding model. All reagents were diluted in HBS-EP^+^ pH 7.4 buffer (0.01 M HEPES, 0.15 M NaCl, 3 mM EDTA, 0.005% Tween-20). The chip was regenerated using a Biacore Regeneration solution (pH 1.7) (GE Healthcare) after each dissociation step.

### ELISA-based measurements of T-CEP binding simultaneously to CD3 and CD28 extracellular domains.

High-protein binding polystyrene ELISA plates (Corning) were coated overnight at 4°C with hIgG1-tagged CD3Ɛδ (Cambridge Biologics) or a human IgG1a-tagged VISTA-IgV domain (negative control) (49) (4 μg/mL) dissolved in PBS. Wells of plates were blocked with 1% w/v BSA (MilliporeSigma) for 1 hour at room temperature. T-CEP (10 μg/mL) was then dispensed in each well and incubated for 1 hour. The wells were washed with PBS, and biotinylated human recombinant hIgG1-tagged CD28 (P10747-1) was added to the wells for 1 hour, then detected using streptavidin-HRP (BioLegend) and TMB (Thermo Fisher Scientific). Human recombinant hIgG1-tagged CD28 was biotinylated using EZ-Link Sulfo-NHS-LC-Biotin (Thermo Fisher Scientific).

### T cell retrieval from human PBMCs.

Human PBMCs were isolated from blood samples of 5 healthy donors using a Ficoll gradient. The recovery of CD3^+^ human T cells was then achieved using an EasySep Human T Cell Isolation Kit (STEMCELL Technologies). T cells were then seeded in a 96-well plate at a density of 5 × 10^5^ cells/mL in X-VIVO 10 (Lonza) Hematopoietic Cell Medium supplemented with 5% fetal bovine serum.

### T cell proliferation and cytokine secretion.

For immobilized T cell activation, 96-well plates were coated with anti-CD3 (OKT3 Bio X Cell) at 4°C overnight. Wells were then washed 3 times with PBS before seeding cells. Soluble anti-CD28 (9.3 Bio X Cell) and soluble activators were added upon seeding. ImmunoCult Human CD3/CD28 TAC soluble activator (STEMCELL Technologies) was used according to the manufacturer’s protocol to deliver anti-CD3 and anti-CD28 in TACs. The estimated concentration of 1.5 μg/mL for the TACs was calculated based on absorbance measurements recorded at 280 nm.

For measuring the expansion of initially proliferating cells, isolated human CD3^+^ T cells were labeled with CFSE following the manufacturer’s protocol (Thermo Fisher Scientific). After 5 days, T cells were harvested and analyzed by flow cytometry (BD LSR II, BD Biosciences). The levels of IL-2, IFN-γ, and TNF-α secreted in the medium were quantified using a LEGENDplex multianalyte flow assay kit (BioLegend).

### 12-Day expansion protocol.

For the 12-day expansion period, cells were directly seeded into a 96-well plate, and the medium was supplemented with 100 IU/mL recombinant human IL-2 (STEMCELL Technologies). Cells were then transferred to 24-well G-rex plates (Wilson Wolf). Aliquots of 10 μL were taken from duplicate wells for counting using a hemocytometer and for flow cytometry except for day 3, when 2 wells representative of each stimulation method were used. The medium containing 100 IU/mL IL-2 was replenished every 3 days. To calculate changes in MFI signals, values recorded for CD25 and CD38 on day 0, before stimulation, were subtracted from the MFI measurements taken every 3 days. The MFI value of the appropriate isotype control was subtracted from the MFI signal recorded for CD27 expression, to calculate the change in CD27-related MFI signals.

### Flow cytometry.

T cells collected from duplicate wells were combined and stained with appropriate antibody conjugates for 30 minutes at 4°C, then washed and resuspended in 30 nM DAPI prepared in PBS. Cell populations were subsequently analyzed by flow cytometry (BD LSR II).

Antibody conjugates, Alexa Flour 647–anti-CD8 (RPA-T8), PE/Cy7–anti-CD4 (OKT4), Alexa Flour 700–anti-CD25 (BC96), FITC–anti-CD38 (HIT2), PE–anti–PD-1 (EH12.2h7), PerCP-Cy5.5–anti–LAG-3 (11C3C65), Dazzle–anti-CD27 (0323), and APC/Cy7–anti-CD45RA (HI100), and appropriate isotype controls were purchased from BioLegend. DAPI was bought from Thermo Fisher Scientific.

### Statistics.

Statistical analyses were performed using Prism 8 (GraphPad). A *P* value of less than 0.05 was considered significant. The statistical significance between more than 2 groups was determined using a 1-way ANOVA or a repeated measures ANOVA with multiple-comparison tests. Data in figures represent mean ± SD.

### Study approval.

Human blood samples were collected with the informed consent of donors for approved use of donors’ blood for research (project identification number 443-2017), carried out in accordance with the guidelines set forth by Sunnybrook Health Sciences Research Ethics Board (Sunnybrook Research Institute, Toronto, Ontario, Canada).

## Author contributions

EM and JG conceptualized the project and wrote the paper. EM performed and designed experiments and analyzed data. AS provided critical guidance for experimental studies.

## Supplementary Material

supplemental data

## Figures and Tables

**Table 1:**
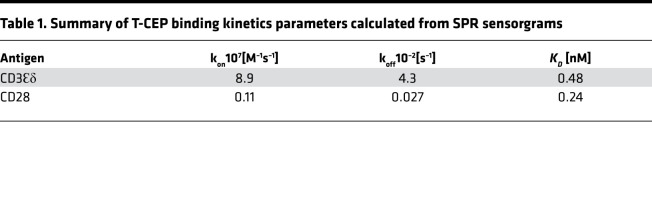
Summary of T-CEP binding kinetics parameters calculated from SPR sensorgrams

## Version 1 11/19/2020

Electronic publication
