# Supplementary material for: A soluble activator that favors the ex vivo expansion of CD8+CD27+ T cells
Source: JCI Insight. 2020 Nov 19;5(22):e141293. doi: 10.1172/jci.insight.141293 (PMC7710272; doi:10.1172/jci.insight.141293)
Supplement: supplemental data [file jciinsight-5-141293-s031.pdf]

Supplemental Figures

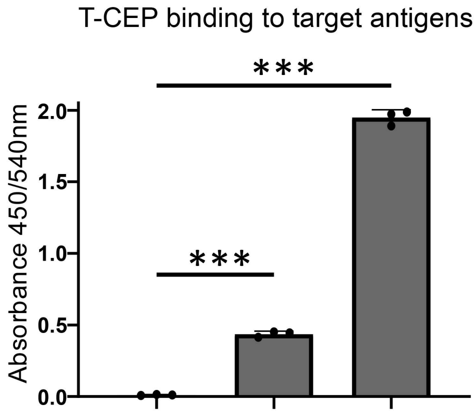

Step 1	T-CEP	T-CEP	T-CEP
Step 2	igG control	rhCD3εδ	rhCD28
Step 3	Anti-hIgG-HRP	Anti-hIgG-HRP	Anti-hIgG-HRP

Supplemental Figure 1: T-CEP binding to target antigens was demonstrated by enzyme-linked immunosorbent assay (ELISA). A sandwich ELISA was carried out by coating T-CEP on high-binding wells (4µg/mL) and target antigens added to wells. The binding of recombinant human CD3εδ-Fc (4µg/mL), recombinant human CD28-Fc (4µg/mL) , and an Fc-matched control (4µg/mL) was detected using an anti-hIgG-HRP conjugate (n=3, one-way ANOVA with Dunnett's test). **Terms:** rhCD3εδ, recombinant human CD3εδ; rhCD28, recombinant human CD28; **Symbols:** *** $P<0.001$.

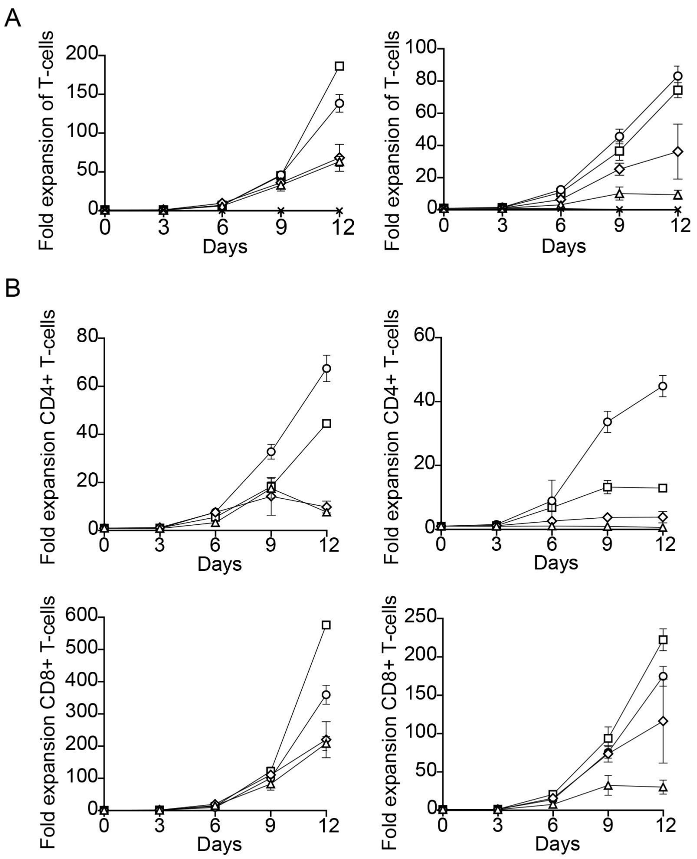

Supplemental Figure 2: The individual expansion profiles of PBMC Donors #4 (left) and #5 (right). For each donor, the number of viable cells were counted in duplicate samples using a hemocytometer following the staining of dead cells with trypan blue for each expansion method tested **(A)** 12-Day expansion using specified stimulation and low level of IL-2. **(B)** $CD4^+$ and CD8+ T-cell expansions of two donors. The fold expansion was calculated based on the starting number of CD4+ and CD8+ T-cells. **Terms:** TACs,Tetrameric antibody complexes; i αCD3, Immobilized anti-CD3; s αCD28, Soluble anti-CD28. **Symbols:** □, T-CEP; ○, TACs; ◇, i αCD3 + s αCD28; △, i αCD3.
